# Nanoparticles of Copper Stimulate Angiogenesis at Systemic and Molecular Level

**DOI:** 10.3390/ijms16034838

**Published:** 2015-03-03

**Authors:** Natalia Mroczek-Sosnowska, Ewa Sawosz, Krishna Prasad Vadalasetty, Monika Łukasiewicz, Jan Niemiec, Mateusz Wierzbicki, Marta Kutwin, Sławomir Jaworski, André Chwalibog

**Affiliations:** 1 Division of Poultry Breeding, Warsaw University of Life Sciences, Ciszewskiego 8, 02-786 Warsaw, Poland; E-Mails: nataliamroczek1@wp.pl (N.M.-S.); monika_lukasiewicz@sggw.pl (M.L.); jan_niemiec@sggw.pl (J.N.); 2 Division of Nanobiotechnology, Warsaw University of Life Sciences, Ciszewskiego 8, 02-786 Warsaw, Poland; E-Mails: ewa_sawosz@sggw.pl (E.S.); mateusz_wierzbicki@sggw.pl (M.W.); marta_prasek@sggw.pl (M.K.); slawomir_jaworski@sggw.pl (S.J.); 3 Department of Veterinary Clinical and Animal Sciences, University of Copenhagen, Groennegaardsvej 3, 1870 Frederiksberg, Denmark; E-Mail: krish@sund.ku.dk

**Keywords:** angiogenesis, copper nanoparticles, chicken embryo, gene expression

## Abstract

Copper is a key element affecting blood vessel growth and muscle development. However, the ions released from Cu salts are toxic. Given their specific physicochemical properties, nanoparticles of Cu (NanoCu) may have different bioactivity and affect the development of blood vessel and muscles in a different manner than Cu salts. The objective of the study was to evaluate the influence of NanoCu on embryo development and angiogenesis at the systemic and molecular level, in experiments using a chick embryo model. Fertilized chicken eggs were divided into a control group, and groups injected with a placebo, CuSO_4_ or NanoCu. Embryo development at the whole body level and molecular indices using an embryo chorioallantoic membrane model were measured during embryogenesis. The present study indicated for the first time that NanoCu have pro-angiogenic properties at the systemic level, to a greater degree than CuSO_4_ salt. The properties of NanoCu were confirmed at the molecular level, demonstrating significant effects on mRNA concentration and on mRNA gene expression of all pro-angiogenic and pro-proliferative genes measured herein.

## 1. Introduction

Copper is a key microelement required by animals and humans. Although the content of Cu in the human body is only about 100 mg it plays an important and multifunctional role as a cofactor of many enzymes [1]. Cu serves as a catalytic and structural cofactor for enzymes that function in energy generation (as a component of cytochrome c oxidase (COX), and is involved in iron acquisition, oxygen transport, cellular metabolism, peptide hormone maturation, blood clotting, signal transduction and a host of other processes [2].

Cu is essentially involved in angiogenesis as a stimulator of angiogenesis, vasculogenesis and endothelial cell migration [3]. Cu levels were found to locally regulate the growth or regression of new blood vessels. Furthermore, Cu affects the expression of structurally diverse but vital angiogenic growth factors such as vascular endothelial growth factor (VEGF), fibroblast growth factor 2 (FGF2), tumor necrosis factor (TNF)-α and platelet-derived endothelial cell growth factor (PD-ECGF) [4]. VEGF is recognized as the key regulator of angiogenesis, and is more efficient than FGF2 in the stimulation of differentiation of endothelial cells [5]. Cu is required for the activation of hypoxia-inducible factor-1 (HIF-1), a major transcription factor regulating the expression of VEGF [6]. As an angiogenic cofactor, Cu is also implicated in new vessel formation by interacting with endothelial and smooth muscle cells, leading to cell migration, invasion, proliferation and the formation of tubular structures [7]. Cu has also been shown to stimulate angiogenesis in chick embryo chorioallantoic membrane (CAM) models [4].

Muscle development is determined mainly during embryogenesis, and consequently the final number of muscle fibers is accomplished in the prenatal and early post hatch periods [8]. Moreover, muscle maturation during embryogenesis is dependent on the development of a vessel network, which provides cells with oxygen and nutrients; Cu may interfere indirectly with the molecular status of muscle maturation during embryogenesis via effects on myoblast determination protein 1 (MyoD1) and paired box protein 7 (Pax7).

At the nanometer scale (1–100 nm) there are collections of atoms or molecules, the properties of which are neither those of the individual constituents nor those of the bulk material. At this scale, many atoms are located on the surface of nanoparticles [9]. The unique bioactivity of nanoparticles together with their very small size influence the properties and behavior of nanoparticles when introduced to an organism, related to transportation, distribution, biochemical activities and molecular responses [10]. As an essential element that is present in very small amounts in the body, Cu is involved in many different processes, playing a dual role in the organism (also being a pro-cancer agent), and may in the form of nanoparticles show different effects. Chen *et al.* [11] reported that the LD_50_ for Cu nanoparticles (size 23.5 nm), Cu particles (size 17 μm) and ions in mice was 413, >5000 and 110 mg/kg body weight, respectively. This result classified both nanoparticles and ions as class 3 (moderately toxic) agents. Given that nanoparticles seem to be less toxic than ions in standard toxicological experiments, they have potential applicability as biological agents, making it important to investigate molecular responses to Cu nanoparticles.

The objective of this study was to evaluate the influence of nanoparticles of copper on angiogenesis at the systemic and molecular level in comparison to Cu salt, in experiments using a chick embryo model.

## 2. Results

Chicken embryo development, at day 3, 6, 9, 12, 15, 18 and 20 of incubation, was compared with the Hamburger–Hamilton normal stages of chicken embryo development. All embryos developed normally and no genetic or other defects were detected. The administration of CuSO_4_ and NanoCu to the chicken embryo by the *in ovo* method did not influence the weight of the body, heart, liver or spleen evaluated at day 20 (Table 1).

**Table 1:** Weight of the body and chosen organs, evaluation according to Hamburger–Hamilton development stages (HH) and mortality of chick embryo at 20 day, after *in ovo* treatment with CuSO_4_ and nanoparticles of Cu (NanoCu).

Indices	Groups			ANOVA	
	Control	CuSO_4_	NanoCu	SEM	*p*-Value
Body Weight	49.5	48.67	49.93	3.672	ns
Liver	1.128	1.001	1.001	0.143	ns
Spleen	0.028	0.026	0.024	0.0061	ns
Heart	0.434	0.382	0.420	0.0391	ns
HH	correct	correct	correct		
Mortality %	8.1	11.2	9.0		

ns = non-significant, *p* < 0.05.

**Figure 1:**
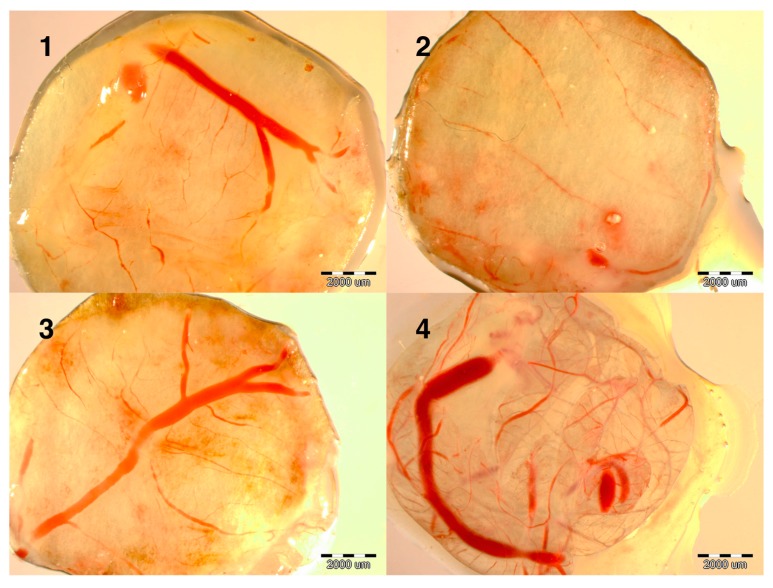
Images of implants maintained on the chicken embryo chorioallantoic membrane (CAM) for 2 days, soaked with: (**1**) control (not soaked); (**2**) control (PBS); (**3**) CuSO_4_; (**4**) NanoCu, evaluated at day 12 of incubation. Scale bars, 2000 µm.

Evaluation of the development of the embryos at day 9 showed that the development of blood vessels was more intense in the NanoCu group than in the CuSO_4_ group and much denser than in the control group (data not shown). To verify this observation experiments were carried out on the induction of angiogenesis. Implants soaked with PBS, CuSO_4_ or NanoCu were placed on the CAM and after 2 days, at day 12 of incubation, the vessel network was observed. Both forms of Cu promoted the development of vessels; however, the number of branches and length of vessels was greater in the NanoCu group compared to the control and the CuSO_4_ group (Table 2, Figure 1).

**Table 2:** The number of branches of blood vessels examined on implants (with diameter 1 cm) incubated on the chick embryo chorioallantoic membrane, after *in ovo* treatment with CuSO_4_ and nanoparticles of Cu (NanoCu).

Indices	Groups			ANOVA	
	Control	CuSO_4_	NanoCu	SEM	*p*-Value
Number of Branches	7.9 ^a^	12.6 ^b^	17.3 ^c^	0.43	0.000
Length of vessels mm	7.3 ^a^	11.5 ^b^	13.2 ^b^	0.97	0.000

^a,b,c^: within rows, means bearing different superscripts differ significantly at *p* < 0.05.

The results concerning *VEGF-A*, *FGF2*, *PCNA*, *MyoD1*, *Pax7* and *COX* gene expression normalized to the *β-actin* (ACTB) gene at the mRNA level indicated that the experimental treatments influenced mRNA synthesis in embryo breast muscle (Table 3).

**Table 3:** The mRNA expression of *VEGF-A*, *FGF2*, *PCNA*, *MyoD1*, *PAX7* and *COX* genes and concentration of DNA and RNA in the samples of pectoral muscles of embryos at day 18 and day 20, after *in ovo* treatment with CuSO_4_ and nanoparticles of Cu (NanoCu).

Indices	Groups			ANOVA	
	Control	CuSO_4_	NanoCu	SEM	*p*-Value
	Day 18				
*VEGF-A*	11.14	13.84	10.87	1.231	ns
*FGF2*	4.12 ^a,b^	2.92 ^b^	5.42 ^a^	0.957	0.012
*PCNA*	0.90	0.73	0.68	0.085	ns
*MyoD1*	0.37 ^a^	0.78 ^b^	0.57 ^a,b^	0.147	0.014
*COX*	4.96	3.15	3.59	0.805	ns
*Pax7*	0.59	0.58	0.65	0.075	ns
DNA	258.7	202.5	249.0	39.18	ns
RNA	2194.2	2291.0	2391.9	186.6	ns
RNA/DNA ratio	8.5	11.3	9.6		
	Day 20				
*VEGF-A*	21.69 ^a^	25.66 ^a^	36.96 ^b^	2.184	0.000
*FGF2*	1.30 ^a^	6.72 ^b^	9.00 ^c^	0.952	0.000
*PCNA*	0.68 ^a^	0.62 ^a^	2.54 ^b^	0.134	0.000
*MyoD1*	1.08 ^a^	3.10 ^c^	1.91 ^b^	0.327	0.000
*COX*	16.81	16.20	18.52	2.085	ns
*Pax7*	0.62 ^a^	0.21 ^b^	0.25 ^b^	0.269	0.000
DNA	249.3	289.9	247.8	43.06	ns
RNA	1182.6 ^a^	1066.3 ^b^	1798.6 ^c^	82.17	0.000
RNA/DNA ratio	4.7	3.7	7.3		

ns = non-significant; ^a,b,c^: within rows, means bearing different superscripts differ significantly at *p* < 0.05.

*VEGF-A* expression did not differ significantly between embryos treated with CuSO_4_ and NanoCu at day 18 but at day 20, expression tended to be higher in the CuSO_4_ group and was significantly higher in the NanoCu group than in the control group (1.8 times higher). The expression of *FGF2* was not significantly affected at day 18 in comparison to the control group; however, NanoCu had a significantly greater effect than CuSO_4_. At day 20, *FGF2* was upregulated by CuSO_4_, and to a greater extent by NanoCu. *FGF2* expression was about five-fold higher in CuSO_4_ and seven-fold higher in the NanoCu group than in the control group. Neither CuSO_4_ nor NanoCu affected the expression of *PCNA* at day 18, but at day 20, expression was significantly increased by NanoCu treatment. When MyoD1 was examined at day 18, the level of expression was significantly higher in embryos treated with CuSO_4_ compared to the control group. At day 20, *MyoD1* expression was significantly higher in the embryos treated with NanoCu and even higher (three-fold higher) in the CuSO_4_ than in the control group. The expression of *COX*, examined at day 18 and 20, was not affected. *Pax7* gene expression was not influenced by experimental treatments at day 18; however, at day 20, it was significantly reduced in the CuSO_4_ and NanoCu groups compared to the control group. The DNA and RNA concentrations in the breast muscle at day 18 were not affected by the treatments, but at day 20 the RNA concentration was lower in the CuSO_4_ group and substantially higher in the NanoCu group compared to the control group.

## 3. Discussion

The results indicated that copper in the form of salt (CuSO_4_) as well as in metal form (Cu°), introduced into chick embryos by *in ovo* injection, did not influence embryo growth or development. Neither form of Cu was harmful when injected as 300 μL of colloid/salt at a concentration of 50 ppm of Cu. These results confirm our earlier findings with chick embryos and growing chicks [12,13]. Moreover, our previous studies indicated that neither Cu nanoparticles nor silver or gold nanoparticles interfere with the growth and development of embryos when used at levels up to 50 ppm [14,15,16].

CuSO_4_ is a source of Cu ions whereas nanoparticles of Cu are a source of particles of metal with a highly bioactive surface and also a small quantity of Cu ions. Interestingly the amount of ions released is strongly dependent on the pH of the environment (compartment of cells), which is also positively correlated with the toxicity of Cu nanoparticles. Nevertheless, at pH = 7 the percentage of Cu ions originating from nanoparticles was 0.1%–0.3% [17]. The physical form (ions *vs.* metal) influences the majority of Cu bio-properties, hence, the key issue of the present investigation was to indicate differences between Cu salt and Cu nanoparticle bioactivities in the process of angiogenesis at the systemic and molecular level.

In our experiment, we observed more intense development of the vessel network (growing outside the chicken embryo until day 15) and increased vascularization of implants maintained on CAM after CuSO_4_ treatment in comparison to the control group. In experiments using the rabbit cornea vascularization model, the application of CuSO_4_ provoked neovascularization [18]. It is known that Cu is involved in angiogenesis, being a pleiotrophic agent that influences numerous mediators of angiogenesis [6,19]. This fundamental mechanism has even been used as a promising anticancer treatment [20]. Our observations confirm this phenomenon.

However, the pro-angiogenic effects observed after NanoCu treatment, which were significantly more potent compared to those produced by CuSO_4,_ have never been reported. Thus, the present study is the first to indicate that nanoparticles of Cu have pro-angiogenic properties at the systemic level, to a greater degree than CuSO_4_ salt.

Gene expression at the mRNA level, measured in samples of breast muscle from the chick embryos, did not unambiguously support these observations. New vessel growth requires the chronological activation of many receptors by numerous ligands, but a critical rate-limiting step in angiogenesis is VEGF signaling [21]. Cu is required for the stimulation of hypoxia-inducible factor-1 (HIF-1), the most important transcription factor regulating the expression of VEGF [20]. VEGF is also responsible for the proper development of the vascular system in skeletal muscles [22], in which gene expression was measured in our experiment. Further, VEGF as an endothelial cell mitogen is involved in angiogenesis as well as vasculogenesis. In our experiment expression of *VEGF-A* was not affected by either treatment at day 18, or by CuSO_4_ treatment at day 20, but at day 20 NanoCu treatment almost doubled the expression of *VEGF-A* compared to the control. VEGF-A is regulated by hypoxia, oxidative stress, growth factors and cytokines [23,24]. However, during embryogenesis in normal physiological conditions, FGF plays a crucial role in VEGF-dependent vasculogenesis. Experiments with epicardial cells, which form vascular tubes, indicated that most of the FGF family of proteins, including FGF2, regulates the tubulogenic response [25]. Interestingly, FGF effects are VEGF dependent, while tubulogenesis induced by VEGF also requires FGF signaling. This interaction between *VEGF-A* and *FGF2* was seen at the level of gene expression at day 20. The highest mRNA expression of *VEGF-A* and *FGF2* was observed in the NanoCu treatment, consistent with vessel network development in the chicken embryo and CAM implant model.

At day 18, however, neither VEGF nor FGF2 expression were significantly influenced by Cu treatments compared to the control group. Nevertheless, the FGF2 mRNA concentration was higher after NanoCu than CuSO_4_ treatment. The number and migration of endothelial cells was significantly increased by FGF2 [26]. FGF2 transmits signals preferentially via tyrosine kinase receptors (FGFR1–4), non-tyrosine kinase receptors and also via cell-surface FGFR-interacting proteins [26]. The spectrum of the interaction of FGF2 is very wide, and CuSO_4_ and NanoCu may interfere with the same or only partly the same signal pathways. NanoCu as metallic particles may be sequestered in different ways than Cu salt, and consequently the signaling properties of the two forms of Cu may differ. It is also possible that the activity of FGF2 during embryogenesis, which might be modulated by CuSO_4_ or NanoCu, is time dependent and that biosignaling by the metallic form (NanoCu) occurs over a longer period compared to CuSO_4_ salt. Furthermore, the reason that expression of FGF2 after NanoCu treatment was higher compared to the CuSO_4_ treatment at the end of embryogenesis, may be a more efficient transport and different signaling pathways of the metallic form. Hence, it will be crucial to investigate the behavior of NanoCu within tissue and cells in future experiments.

FGF2 activity is observed from early phases of embryogenesis and is highly involved in cell proliferation [27,28], and consequently is a strong PCNA inducer [29]. This was reflected in the expression of PCNA after NanoCu treatment at day 20. In comparison to CuSO_4_, NanoCu significantly increased *PCNA* expression—a key marker of cell division. However, this was not observed at day 18, pointing to the possibility that NanoCu extend the period of predominance of hyperplasia over hypertrophy at the end of embryogenesis.

To identify further molecular effects on breast muscle development after treatment with different forms of copper two other FGF2-dependent genes were examined: *MyoD1* and *Pax7*. MyoD1, unlike FGF2, activates mechanisms of skeletal muscle lineage determination and differentiation [30]. Myogenic regulatory factors (including MyoD1) in the embryo muscle are markers of cell differentiation, inducing the synthesis of myogenin, which leads to the suppression of cell proliferation. This process increases at the end of embryogenesis but at the end of embryo development the proliferation of muscle cells slows down and almost ceases [31]. In the current study treatment with CuSO_4_ increased *MyoD1* expression at day 18 two-fold and at day 20 three-fold in comparison to the control. It is possible that CuSO_4_ might increase cell differentiation by increasing the vessel network and transport of oxygen and nutrients to muscle cells. However, NanoCu increased the *MyoD1* mRNA concentration only at day 20 and to a lower degree in comparison to CuSO_4_. This was also confirmed by the reduced expression of *Pax7*, which in contrast to the anti-proliferative MyoD1, is a marker of proliferation of satellite cells. These differences between CuSO_4_ and NanoCu activity also suggest that the bioactivity of NanoCu might be more prolonged.

In the light of examination of vessel network development at the systemic level, the present results show that NanoCu stimulate angiogenesis more than CuSO_4_. Moreover, the molecular responses demonstrate that NanoCu as well as CuSO_4_ affect the mRNA expression of genes involved in angiogenesis and cell proliferation. This may indicate that the level of Cu stored in the egg is insufficient, notably at the end of embryogenesis. Furthermore, NanoCu have a stronger effect on mRNA gene expression than CuSO_4_, which may point to a different mechanism of signaling, as well on more efficient sequestration within muscle cells.

## 4. Experimental Section

### 4.1. Experimental Design

Chicken eggs from 37-week-old Ross × Ross 308 hens, obtained from a commercial certificated hatchery, were stored in a refrigerator (10 °C) for 1–3 days and then placed in an incubator. The eggs were randomly divided into four groups (4 × 100 eggs): no injection (control), injected with phosphate buffered saline (PBS) as a placebo, or injected with solutions of CuSO_4_ (CuSO_4_) or hydrocolloid of nanoparticles of copper (NanoCu). At day 1 of incubation, the eggs were weighed (58 ± 1.28 g) and 0.3 mL of experimental fluids was injected under sterile conditions into the air sac, using 27-gauge, 20-mm needles. Immediately after the injection, the hole was sealed with sterile tape and the eggs were placed in an incubator. The eggs were incubated for 18 or 20 days under standard conditions (temperature 37.8 °C, humidity 55%, turned once per hour for the first 18 days, and at a temperature of 37 °C and humidity 60% from day 20).

At day 3, 6, 9, 12, 15, 18 and 20 of incubation eggs were randomly selected from each group and six embryos from each group were dissected and examined. The developmental status of the chick embryos was compared with the developmental stages described by [32]. The network of blood vessels in the chorioallantoic membrane (CAM) was evaluated at day 9 and day 12. At day 18 six samples of the breast muscle were collected in RNAlater^®^ ribonucleic acid (RNA) stabilization solution (Applied Biosystems/Ambion, Austin, TX, USA) for mRNA expression analysis. At day 20 the embryos were weighed and decapitated, the liver, heart, spleen were weighed and the breast muscles were prepared for mRNA expression analysis.

### 4.2. Experimental Solutions

CuSO_4_ was obtained from Sigma Aldrich, St. Louis, MO, USA and was dissolved in ultra pure water to a concentration of 50 ppm of Cu. The hydrocolloid of NanoCu, at a concentration of 50 ppm, was purchased from Nano-Tech, Warsaw, Poland and was produced by a non-explosive high voltage method from high purity metals (99.9999%) and high purity demineralized water.

**Figure 2:**
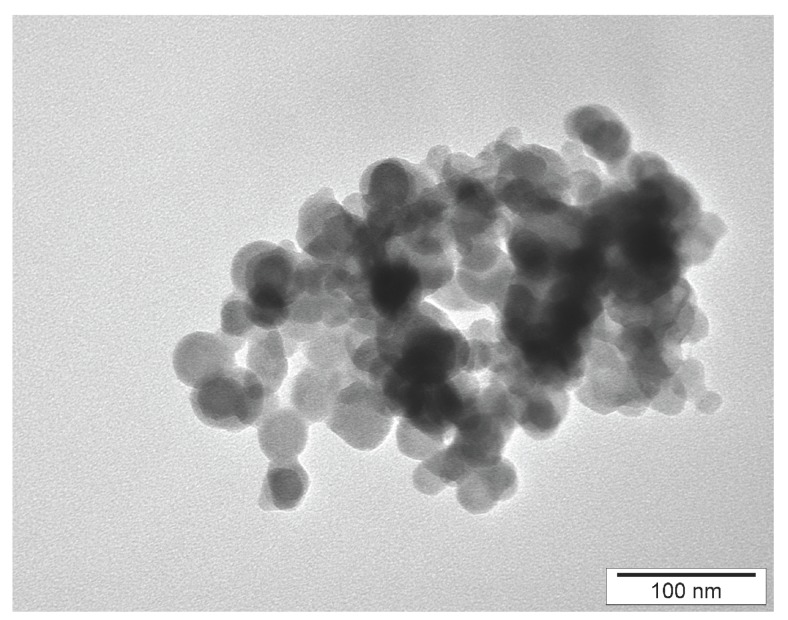
Transmission electron microscopic image of Cu nanoparticles.

The Zeta potential of hydrocolloids was measured using a Zetasizer Nano-ZS90 (Malvern Instruments Ltd., Malvern, UK): the Zeta potential of NanoCu was −28.1 indicating a stable solution. The shape and size of NanoCu were visualized using a JEM-2000EX transmission electron microscope (TEM: JEOL Ltd., Tokyo, Japan) at 80 kV (Figure 2). The nanoparticles were spherical and rarely elliptical, rounded, with no sharp edges. The diameter of nanoparticles was on average 37.3 nm and varied from 15 to 70 nm.

### 4.3. Gene Expression at the mRNA Level

Gene expression at the mRNA level was measured using the quantitative polymerase chain reaction method (qPCR). The tissue dissected from the breast muscle was homogenized in TRIzol^®^ Reagent (Life Technologies, Naerun, Denmark), and total RNA was extracted according to the manufacturer’s instructions. The RNA samples were purified using the SV Total RNA Isolation System (Promega Corporation, Madison, WI, USA) and quantified using a NanoDrop ND 1000 spectrophotometer (Thermo Fisher Scientific, Waltham, MA, USA). Using reverse transcriptase with oligo (dT) (Promega) and random primers (TAG Copenhagen A/S, Copenhagen, Denmark), 2 mg of total RNA was reverse transcribed, after which real-time PCR was performed with complementary DNA and gene-specific primer pairs (TAG, Copenhagen A/S, Copenhagen, Denmark) mixed with LightCycler^®^ 480 SYBR Green I Master mix (Roche Applied Science, Penzberg, Germany) in a LightCycler^®^ 480 real-time PCR system (Roche Applied Science, Penzberg, Germany). The following primers were used: *FGF2* (forward: GGCACTGAAATGTGCAACAG, reverse: TCCAGGTCCAGTTTTTGGTC), *VEGF-A* (forward: TGAGGGCCTAGAATGTGTCC, reverse: TCTTTTGACCCTTCCCCTTT), *PCNA* (forward: TGCACGCATTTGTAGAGACC, reverse: AGTCAGCTGGACTGGCTCAT), *MyoD1* (forward: CGGCGGCTCAGCAAGGTCAAC, reverse: CGGCCCGCTGTACTCCATCATG), *COX* (forward: AGGATTCTATTTCACAGCCCTACAAG, reverse: AGACGCTGTCAGCGATTGAGA), *Pax7* (forward: CCAGTAGAGACAGGCCAAGC, reverse: GGAGTTGGGAAGGAGTAGGG) and *ACTB* (forward: GTCCACCTTCCAGCAGATGT, reverse: ATAAAGCCATGCCAATCTCG). For each complementary DNA, the reaction was performed in triplicate. For analyses, relative quantification was conducted using *ACTB* as the housekeeping gene.

### 4.4. Angiogenesis Assay

Angiogenesis (state of development of the vascular network) was evaluated using the chick CAM implantation method according to [33,34]. The CAM method could be performed till day 12 of incubation because only in this period vessels grow outside the embryo body. Eggs (3 groups × 20 eggs) were incubated as described above. On the 3rd day of incubation a 1 cm^2^ window in the shell was opened and 3 mL of albumen was removed. The window was sealed with sterile aluminum foil and the eggs were placed back in the incubator. At day 9 of incubation the windows were opened and sterilized filter paper rings, loaded with experimental solutions (CuSO_4_ or NanoCu) or PBS as a placebo were placed over the developing CAM. The implants were pre-treated with 3 mg/mL of hydrocortisone sodium succinate (Sigma, St. Louis, MO, USA) and air dried under sterile conditions. The concentration of Cu in CuSO_4_ and NanoCu was 50 ppm. The windows were then resealed, and the eggs were incubated for a further 48 h. At day 12 of incubation, the implants were removed from the eggs [34] and the morphology of blood vessel formation around the implants was observed using a stereomicroscope under 12.5-fold magnification (SZX10, CellD software version 3.1, Olympus Corporation, Tokyo, Japan). Photos were analyzed using CellSens Dimension Desktop version 1.3 (Olympus Corporation). The vessel network (length of vessels and number of branches) present on the implants was evaluated according to [34].

### 4.5. Statistical Methods

Data was normally distributed and the results were analyzed by analysis of variance computed with the least squares method using statistical software SPSS 19.0 PL for Windows (SPSS Inc., Chicago, IL, USA), at a significance level of *p* ≤ 0.05.

## 5. Conclusions

We demonstrated that *in ovo* administration of Cu as CuSO_4_ salt or as NanoCu at a concentration of 50 ppm had no negative effects on embryo development. Both treatments increased vascularization of implants maintained on CAM, but the pro-angiogenic effects of NanoCu were more potent compared to those produced by CuSO_4_. The properties of NanoCu were confirmed at the molecular level, demonstrating significant effects on mRNA concentration and on mRNA gene expression of all pro-angiogenic and pro-proliferative genes measured herein.
